# Tumor microenvironment and key signaling pathways in breast cancer progression and therapy resistance: A review

**DOI:** 10.17305/bb.2026.13708

**Published:** 2026-02-27

**Authors:** Rui Li, Yujing Wang, Min Xie

**Affiliations:** 1 Department of Laboratory Medicine, The Third People’s Hospital of Chengdu, The Affiliated Hospital of Southwest Jiaotong University, Chengdu, China; 2 Department of Laboratory Medicine, The People’s Hospital of Leshan, Leshan, China

**Keywords:** Breast cancer, tumor microenvironment, cell signaling

## Abstract

Breast cancer progression is influenced not only by intrinsic tumor alterations but also by reciprocal interactions with the tumor microenvironment (TME), a complex ecosystem comprising fibroblasts, immune and endothelial cells, adipocytes, extracellular matrix components, soluble mediators, and extracellular vesicles. This review synthesizes recent basic and translational research on how TME-derived signals activate dysregulated signaling pathways, including the phosphoinositide 3-kinase/protein kinase B/mechanistic target of rapamycin (PI3K/AKT/mTOR), transforming growth factor beta/SMAD (TGF-β/SMAD), Janus kinase/signal transducer and activator of transcription (JAK/STAT), mitogen-activated protein kinase/extracellular signal-regulated kinase (MAPK/ERK), Wingless-related integration site/beta-catenin (Wnt/β-catenin), Notch, Yes-associated protein/transcriptional co-activator with PDZ-binding motif (YAP/TAZ), and nuclear factor kappa B (NF-κB). These pathways promote key processes such as invasion, angiogenesis, adaptation to hypoxia, epithelial–mesenchymal transition, immune evasion, cancer stemness, and therapy resistance. We emphasize convergent findings that indicate the feedback loop between tumor cells and the TME sustains plasticity and drug-tolerant states. Additionally, we summarize emerging therapeutic strategies, including stromal and extracellular matrix normalization, immunotherapy combinations, pathway-targeted inhibitors, and nanotechnology-enabled drug delivery. A comprehensive understanding of TME–signaling crosstalk is crucial for overcoming therapeutic resistance in breast cancer.

## Introduction

Breast cancer represents a significant public health challenge, accounting for a substantial proportion of cancer-related mortalities globally [1]. This disease is highly heterogeneous and is categorized into distinct subtypes—namely, estrogen receptor (ER)/progesterone receptor (PR)-positive, HER2-positive, and triple-negative breast cancer (TNBC)—based on specific molecular expression patterns, including the amplification of ER, PR, and human epidermal growth factor receptor 2 (HER2) [2]. Traditionally, clinical cancer researchers have focused on cancer cell experiments that examine cellular metabolism, proliferation mechanisms, genetic alterations, and responses to monotherapy interventions [3, 4]. However, it is increasingly recognized that tumor biology cannot be fully understood through a singular approach, as it undergoes dynamic changes within the complex tumor microenvironment (TME) [5]. The TME encompasses the ecosystem surrounding tumor cells, comprising cellular components such as stromal cells, immune infiltrates, the extracellular matrix, vascular and lymphatic networks, and a diverse array of non-cellular mediators, including growth factors, cytokines, metabolic byproducts, and extracellular vesicles [6].

**Table 1:** Key cellular components of the breast tumor microenvironment: Functions, markers, and therapeutic opportunities

**Cell type**	**Functions in tumor microenvironment**	**Representative markers**	**Therapeutic opportunities / strategies**	**References**
Cancer-associated fibroblasts (CAFs)	ECM remodeling; cytokine and chemokine secretion; promotion of invasion, therapy resistance, and immune exclusion	α-SMA, FAP, PDGFR-β	TGF-β pathway inhibitors; FAP-targeted therapies; LOX/LOXL2 and FAK inhibitors to reduce stiffness	[11–14]
Tumor-associated macrophages (TAMs)	Angiogenesis; ECM remodeling; immune suppression; support for metastasis	CD68, CD163, CD206	CSF1R inhibitors; CD47–SIRPα blockade; TAM reprogramming (M2→M1) agents	[21, 22]
T cells (CD8^+^ T cells, Tregs)	CD8^+^: cytotoxic anti-tumor immunity; Tregs: immune suppression and tolerance	CD8, FOXP3	Key therapeutic targets include immune checkpoint inhibitors (PD-1/PD-L1), TGF-β inhibitors, and CXCL12/CXCR4 antagonists	[22, 25, 34]
Myeloid-derived suppressor cells (MDSCs)	T-cell suppression; ROS/arginase-driven immune dysfunction; metastasis facilitation	CD11b, CD33, ARG1	MDSC-targeting agents; STAT3/PI3K inhibitors; blockade of MDSC recruitment signals	[32, 123]
B cells (effector and regulatory)	Antibody production; antigen presentation; IL-10–rich regulatory phenotypes	CD19, CD20, IL-10	B-cell modulation; cancer vaccines; targeting regulatory B-cell signals	[124]
Natural killer (NK) cells	Innate cytotoxicity; often functionally exhausted in TME	CD56, NKp46	Cytokine-based support; NK checkpoint modulation; TGF-β blockade	[125, 126]
Endothelial cells	Angiogenesis; formation of hypoxic gradients; stem-like niche signaling	CD31, VEGFR2	Anti-angiogenic agents; vascular normalization strategies	[127]
Pericytes	Vessel stabilization; control of extravasation and metastasis	NG2, PDGFR-β	PDGF signaling modulation; agents promoting vessel normalization	[128]
Adipocytes/ASCs	Lipid transfer to tumor cells; adipokine secretion; metabolic and inflammatory signaling; ASC conversion to CAF-like cells	Leptin, adiponectin, PPARγ	Metabolic inhibitors; targeting leptin/JAK-STAT pathways; obesity-associated TME modulation	[50, 129]

Abbreviations: ARG1: Arginase 1; ASC: Adipose-derived stromal cells; α-SMA: Alpha-smooth muscle actin; CAF: Cancer-associated fibroblast; CXCR4: C-X-C chemokine receptor 4; CXCL12: C-X-C motif chemokine ligand 12; CSF1R: Colony-stimulating factor 1 receptor; ECM: Extracellular matrix; FAP: Fibroblast activation protein; FAK: Focal adhesion kinase; FOXP3: Forkhead box protein P3; IL: Interleukin; JAK–STAT: Janus kinase–signal transducer and activator of transcription; LOX: Lysyl oxidase; MDSC: Myeloid-derived suppressor cells; PD-1/PD-L1: Programmed cell death protein 1/programmed death-ligand 1; PDGF: Platelet-derived growth factor; PDGFR-β: Platelet-derived growth factor receptor beta; PPARγ: Peroxisome proliferator-activated receptor gamma; TGF-β: Transforming growth factor-beta; ROS: Reactive oxygen species; SIRPα: Signal-regulatory protein alpha; STAT3: Signal transducer and activator of transcription 3; PI3K I: Phosphoinositide 3-kinase 1; TAM: Tumor-associated macrophage; TME: Tumor microenvironment; Tregs: Regulatory T cells; VEGFR2: Vascular endothelial growth factor receptor 2.

In breast cancer, the spatial arrangement, composition, and functional interactions between the cellular and non-cellular components of the TME significantly influence disease outcomes and therapeutic responses. For instance, the molecular signaling networks within tumor cells, signal transduction pathways, and intercellular interactions are increasingly recognized as critical mediators of TME-driven effects [6]. Within the TME, alterations in signaling cascades, such as phosphatidylinositol 3-kinase/AKT/mammalian target of rapamycin (PI3K/AKT/mTOR), mitogen-activated protein kinase/extracellular signal-regulated kinase (MAPK/ERK), transforming growth factor-β-SMAD (TGF-β-SMAD), wingless-related integration site/β-Catenin (Wnt/β-Catenin), and nuclear factor kappa-light-chain-enhancer of activated B cells (NF-κB), which are mechanistically significant in tumor cells, are fundamentally influenced by the TME through immune-infiltrating cytokines, growth factors, metabolic reprogramming derivatives, extracellular vesicles, and extracellular matrix remodeling [7]. This bidirectional crosstalk initiates sequential biological processes that contribute to oncogenic features, including tumor development, angiogenesis, adaptation to hypoxia, epithelial-mesenchymal transition (EMT), immune evasion, tumor invasion, metastatic niche formation, and therapeutic resistance [7, 8]. This review aims to provide a comprehensive overview of the basic science landscape, focusing on the interconnected interactions between cellular and non-cellular components of the TME, and highlighting how TME-signal interactions are associated with malignant phenotypes and the pathogenesis of breast tumors.

## The tumor microenvironment components in breast cancer

### Cellular components of the breast tumor microenvironment

In breast cancer, the TME constitutes a dynamic and heterogeneous multicellular ecosystem characterized by direct interactions between malignant epithelial cells and a diverse array of non-malignant components, including immune cell populations, fibroblasts, adipose stromal cells, and endothelial cells. The interactions among these cellular components in the TME and the metabolic reprogramming network directly influence tumor initiation, progression, metastasis, and resistance to treatment [9, 10]. Table 1 outlines the major cellular components of the breast TME, detailing their functions, surface or secretory markers, and promising therapeutic approaches aimed at targeting these cells.

**Figure 1.**
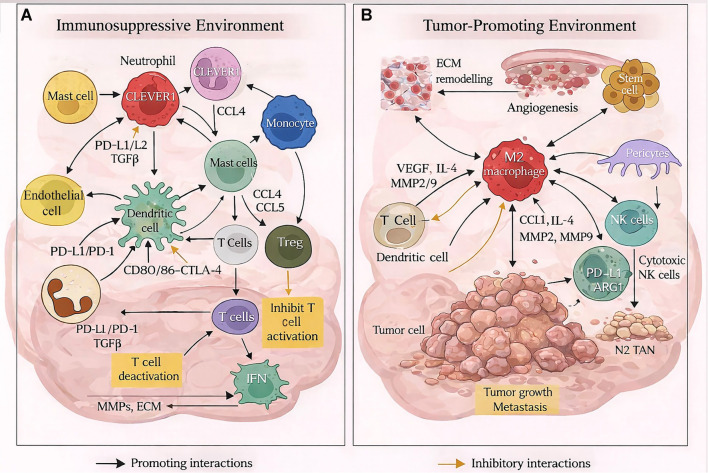
**Immune regulatory and tumor-promoting interactions of immune cell components within the breast tumor microenvironment.** (A) Immunosuppressive environment: This panel illustrates the immune regulatory networks that counteract antitumor immunity. Tumor-associated macrophages, particularly CLEVER1+ macrophages, interact with cellular components of the breast TME, leading to the release of immunosuppressive mediators such as TGF-β, PD-L1/PDL2, and chemokines CCL4 and CCL5. These mediators promote the recruitment and activation of regulatory T cells. Antigen presentation by dendritic cells through CD80/CD86-CTL4 and PD-1 pathways results in T-cell exhaustion and diminished IFN-γ production. Reciprocal signaling between immune and stromal cells increases MMPs activity, facilitates ECM remodeling, promotes immune evasion, and contributes to tumor persistence. (B) Tumor-promoting environment: This panel outlines the mechanisms by which M2 macrophages foster tumor progression and metastasis. M2 macrophages secrete tumor-promoting mediators, including chemokine CCL1, MMP2, MMP9, IL-4, and VEGF, thereby supporting cancer stem cell maintenance, promoting angiogenesis, and facilitating ECM degradation and tumor invasion. The interactions among M2 macrophages, pericytes, and stromal components enhance vascularization and nutrient supply. Additionally, N2 TANs support tumor growth, while ARG1 and PD-L1 inhibit NK cell cytotoxicity, resulting in decreased immune-mediated tumor clearance. The bidirectional cross-talk among immune, stromal, and tumor cells establishes reinforcing feedback loops that promote tumor progression. Black arrows indicate activating interactions, yellow arrows denote inhibitory signaling, and bidirectional arrows represent reciprocal regulation. Abbreviations: ARG1: Arginase-1; CCL: C–C motif chemokine ligand; CLEVER1: Common lymphatic endothelial and vascular endothelial receptor-1; CTLA4: Cytotoxic T-lymphocyte-associated protein 4; DC: Dendritic cell; ECM: Extracellular matrix; IFN-γ: Interferon gamma; IL: Interleukin; MMP: Matrix metalloproteinase; NK cell: Natural killer cell; N2 TAN: N2 tumor-associated neutrophil; PD-1: Programmed cell death protein 1; PD-L1/PD-L2: Programmed death-ligand 1/2; TAM: Tumor-associated macrophage; TGFβ: Transforming growth factor beta; TME: Tumor microenvironment; Treg: Regulatory T cell; VEGF: Vascular endothelial growth factor.

#### Cancer-associated fibroblasts

Cancer-associated fibroblasts (CAFs) are the predominant stromal cell types within the breast TME and perform multiple functions that influence tumor biology. These cells can originate from various sources, including bone marrow-derived mesenchymal stem cells, resident fibroblasts, or EMT of cancer cells. CAFs can exhibit activated malignant phenotypes, characterized by the overexpression of alpha-smooth muscle actin (α-SMA), platelet-derived growth factor receptor-β (PDGFR-β), and fibroblast-activated protein [11, 12].

Activated CAFs release a variety of soluble factors, including matrix metalloproteinases (MMPs), transforming growth factor-β (TGF-β), C-X-C motif chemokine ligand 12 (CXCL12), hepatocyte growth factor (HGF), and IL-6, which promote tumor cell proliferation, invasion, and stemness [13, 14]. Additionally, activated CAFs contribute to tumor aggressiveness and extracellular matrix (ECM) remodeling through collagen deposition and cross-linking, thereby increasing tissue stiffness and activating the integrin/FAK/YAP/TAZ (integrin/focal adhesion kinase/Yes-Associated Protein/TAZ signaling pathway) [15].

Single-cell RNA sequencing analyses have identified different CAF subtypes in breast tumors [16]. Among these, myofibroblastic CAFs (myCAFs) are associated with ECM stiffening, while inflammatory CAFs (iCAFs) promote angiogenesis and immune suppression within the TME by producing cytokines. Antigen-presenting CAFs modulate T lymphocyte activation [17, 18]. These heterogeneities result in pro- and anti-tumorigenic differentiation within the TME, serving as critical determinants of therapeutic response.

#### Immune cells

The immune cell composition within the breast TME exhibits substantial flexibility and spatial heterogeneity, contributing to the balance between anti-tumor immunity and tumor-associated inflammation (Figure 1). This diversity is a crucial determinant of prognosis and therapeutic outcomes in patients with breast cancer [19, 20].

Tumor-associated macrophages (TAMs), particularly those exhibiting an activated M2-like phenotype, constitute a significant proportion of immune cells in the TME. These macrophages secrete IL-10, TGF-β, VEGF (vascular endothelial growth factor), and EGF (epidermal growth factor), promoting angiogenesis, immune suppression, and matrix remodeling in breast tumors [21, 22]. TAMs play a dual role within the TME; increased infiltration of M2-like TAMs correlates with poor prognosis, chemotherapy resistance, and blockade of immune checkpoints. Conversely, M1-like TAMs can enhance pro-inflammatory responses and exhibit anti-tumor activity [23, 24]. Myeloid-derived suppressor cells (MDSCs) produce arginase-1 and reactive oxygen species, attenuating the immune response and facilitating tumor metastasis.

T lymphocytes (T cells) are the second most abundant immune cell type within the breast TME [25]. CD4+ T lymphocytes, including various T helper subtypes (e.g., Th1, Th2, and Th17), orchestrate adaptive immune responses in the TME [26, 27]. Th1 cells enhance anti-tumor immunity by producing interferon gamma (IFN-γ), increasing CD8+ T cytotoxic cells, and activating TAMs [28]. In contrast, Th2 and Th17 cells can promote tumor progression by recruiting eosinophils and neutrophils, respectively, amplifying chronic inflammation through IL-4/IL-13 and IL-17, which upregulate pro-angiogenic factors such as VEGF [26, 29, 30]. An increased infiltration of Th17 cells has been observed in association with advanced TNBC staging and a higher likelihood of metastasis [26, 29]. Regulatory T cells (Tregs), characterized by *Forkhead Box Protein P3* (*FOXP3*) expression, are key mediators of immune evasion [31]. They create a tolerogenic environment within breast tissue by suppressing TGF-β, IL-10, cytotoxic T lymphocyte antigen (CTLA), and effector molecules that inhibit cell proliferation, thereby shielding tumors from immunologic surveillance. Increased infiltration of Tregs has been linked to poor survival in HER2-positive and TNBC patients [28]. In conjunction with Tregs, MDSCs, including granulocytes and monocytes, also contribute to immunosuppression by producing arginase-1 and inducing oxidative/nitrosative stress [32, 33]. In breast tumors, MDSCs facilitate metastasis by remodeling the pre-metastatic niche and correlate with advanced disease, endocrine resistance, and responses to immunotherapy [32]. CD8+ T cells are responsible for eliminating tumor cells; however, their efficacy is often diminished due to prolonged antigen exposure and the programmed cell death protein 1/programmed death-ligand 1 (PD-1/PD-L1) inhibitory axis [34]. Conversely, CAFs can release TGF-β and cytokines, such as C–C chemokine ligands (CCL17/CCL22), which result in the accumulation of Tregs within the TME, suppressing CD8+ T cell activity, along with the presence of functionally exhausted natural killer (NK) cells within the breast TME [25, 35]. B lymphocytes possess the potential to either produce anti-tumor antibodies or maintain a regulatory phenotype characterized by IL-10 production. The distribution of immune cells within the TME is strongly associated with responses to immunotherapy [36–38].

#### Endothelial cells and pericytes

Vascular endothelial cells form the structural foundation of blood vessels and play a crucial role in tumor angiogenesis, nutrient delivery, and metastasis [39]. The aberrant proliferation of malignant cells leads to hypoxia within the TME. The activation of hypoxia-inducible factor-1α (HIF-1α) in stromal and tumor cells increases the secretion of vascular endothelial growth factor (VEGF), thereby perpetuating a cycle of hypoxia and angiogenesis in breast tumors [40, 41]. Although HIF-1α activation enhances VEGF production, the concomitant increase in VEGF, angiopoietin-2, and basic fibroblast growth factor (bFGF) results in abnormal neovascularization characterized by disorganization, increased permeability, and inadequate perfusion. Within the TME, endothelial cells not only regulate oxygen and nutrient gradients but also support cancer stem-like cells by activating signaling pathways such as Notch, Wnt, and PI3K [42]. Additionally, endothelial cells can release exosomes containing microRNAs and other signaling molecules (e.g., growth factors) that influence tumor biology [43].

Pericytes, which are mural cells, stabilize capillaries, promote endothelial cell survival, and modulate vascular maturation by participating in the pericyte-derived PDGF/angiopoietin signaling pathway. During tumor progression, pericytes may detach or lose functionality, exacerbating vessel permeability and facilitating metastasis [39].

#### Adipocytes and adipose stromal cells

Adipocytes are essential components of breast tissue that can undergo metabolic and phenotypic reprogramming, transforming into cancer-associated adipocytes (CAAs) [44]. Typically, CAAs exhibit tumor-promoting effects by increasing lipolysis and secreting inflammatory cytokines (e.g., IL-6 and tumor necrosis factor-alpha (TNF-α)) and adipokines (e.g., leptin, resistin, and adiponectin) into the TME. Depending on the types of adipokines present, breast tumors may progress through proliferation, angiogenesis, and metastasis via leptin signaling, which influences the JAK/STAT3 (Janus kinases (JAKs), signal transducer and activator of transcription proteins (STATs)) and PI3K/AKT signaling pathways, or exert tumor-suppressive effects through adiponectin. CAAs can also provide free fatty acids through lipolysis, oxidative metabolism, and activation of peroxisome proliferator-activated receptors (PPAR) and mTOR signaling pathways, thereby supporting the survival of tumor cells in a nutrient-deprived TME [45, 46]. CAAs and adipose-derived stromal cells contribute to the metabolic and paracrine reprogramming of CAFs. This metabolic interplay between tumor cells and adipocytes is a significant factor in tumor aggressiveness and drug resistance, particularly in ER positive patients [47, 48].

#### TME heterogeneity across solid tumors: Distinctions in breast cancer

While many TME components (e.g., CAFs, TAMs, etc.) are shared across various solid tumors, the breast TME exhibits unique compositional characteristics due to its mammary gland origin [45, 49]. These differences influence tumor progression, immune landscapes, and therapeutic responses, presenting unique challenges and opportunities for breast cancer treatment [50]. For example, the breast cancer TME is significantly enriched with adipocytes and adipose stromal cells, which predominantly produce lipid-associated biomolecules, including adipokines and leptin [45]. Vascularization in breast tumors also differs, as they are typically leaky, sometimes responsive to anti-VEGF monotherapy (e.g., bevacizumab), and exhibit low levels of hypoxia [51, 52]. These distinctions highlight breast-specific opportunities, such as the potential for adipokine pathway inhibitors (e.g., leptin antagonists) or hormone-TME synergies in ER positive patients.

### Non-cellular components of the breast tumor microenvironment

Beyond its cellular heterogeneity, the breast TME encompasses a variety of non-cellular components, including the ECM, soluble factors (e.g., cytokines, growth factors, metabolites, and oxygen gradients), and extracellular vesicles (e.g., exosomes). These components create a heterogeneous biochemical, mechanical, and metabolic environment that significantly influences cancer development.

#### Extracellular matrix remodeling and stiffness

The majority of non-cellular components in the breast TME consist of ECM proteins, which provide a structural network composed of collagens, elastin, laminins, glycoproteins, and proteoglycans. Stromal fibroblasts and CAFs secrete matrix metalloproteinases (MMP2, MMP9, and MMP12) and lysyl oxidase enzymes that significantly affect the ECM, leading to dynamic remodeling characterized by increased collagen-1 cross-links, fibronectin, and laminin. Higher collagen density within the TME is associated with enhanced EMT, tumor invasiveness, and metastasis [53, 54]. Furthermore, the ECM serves as a dynamic reservoir of biochemical compounds, allowing for the release of growth factors that promote cell proliferation and angiogenesis [55].

#### Soluble components

Cytokines, chemokines, and growth factors are soluble mediators within the breast TME that contribute to tumor progression [56]. In breast tumors, pro-tumorigenic cytokines (IL-6, IL-1, and IL-8) and TNF-α activate the JAK/STAT and NF-κB signaling pathways, thereby triggering chronic inflammation, tumor stemness, and survival. CAFs release chemokines such as CXCL12, promoting immune cell recruitment, angiogenesis, and the formation of metastatic niches through the CXCL12/CXCR4 axis. CXCL12 can be secreted into the TME by endothelial cells, fibroblasts, and stromal cells, binding to CXCR4 and activating downstream signaling pathways, including PI3K/AKT, NF-κB, and MAPK/ERK pathways [57]. Overexpression of *CXCL4* has been observed in many breast tumor cells and is associated with poor prognosis in breast cancer patients. Conversely, activation of the CXCL12/CXCR4 axis can induce the overexpression of *VEGF* and the differentiation of endothelial progenitors, indicating hypervascularization and aggressive breast tumors [58]. Other soluble components within the TME include growth factors (e.g., TGF-β, HGF, VEGF, FGF, and PDGF) released by tumor cellular components, which act as paracrine regulators of tumor transformation to pro-metastatic niches. Increased serum levels of IL-6 and TGF-β have been associated with poor prognosis and resistance to therapy [59].

The tumor microenvironment is metabolically heterogeneous due to tumor diversity, rapid cellular turnover, aberrant vascularization, and insufficient perfusion. These conditions lead to inadequate blood flow to tumor tissue, resulting in a hypoxic environment that significantly alters tumor biology. In response to hypoxia, HIF-1α and HIF-2α increase the expression of angiogenic factors (e.g., VEGF), induce anaerobic glycolysis (via glucose transporter 1 (GLUT1) and lactate dehydrogenase (LDH)), and activate EMT-associated genes (e.g., *SNAIL* and TWIST) [60]. Although hypoxia-dependent metabolic reprogramming facilitates the “Warburg effect” within breast tumors, it can also promote the production of lactate by stromal cells such as CAFs, thereby regulating energy balance in a “reverse Warburg effect” manner. Conversely, lactate accumulation within the TME acidifies the environment, promotes M2 macrophage polarization, and suppresses the function of cytotoxic lymphocytes. Another critical aspect of metabolic reprogramming under hypoxia is nutrient deprivation, which affects autophagy and the mTOR signaling pathway, thereby supporting cancer cell survival under stress [61, 62].

#### Extracellular vesicles in the breast TME

Extracellular vesicles, primarily exosomes, are small micro-vesicles (30–150 nm in diameter) that originate from late endosomes of parental cells and are found within the extracellular matrix. These vesicles contain bioactive compounds such as nucleic acids, proteins, signaling molecules, and lipids, enabling them to facilitate intracellular communication within the breast TME [63]. Exosomes derived from tumor cells can contribute to various biological processes. For instance, these nanoparticles can deliver oncogenic microRNAs (e.g., *miR-221* and *miR-*21) to cellular components in the TME (e.g., CAFs and macrophages), promoting M2 polarization and CAF activation. Exosomes containing PD-L1 suppress T lymphocyte activity, facilitating immune evasion. Conversely, exosomes originating from stromal and immune cells can influence angiogenesis and modulate drug sensitivity. Hypoxia enhances exosome production through HIF-1α-regulated Rab GTPases, thereby increasing the release of pro-metastatic vesicles. Due to their stability and detectability in biofluids, exosomes are being explored as biomarkers for early cancer detection and vehicles for targeted therapeutic delivery [63–65].

## Key molecular signaling pathways and tumor microenvironment

The dynamic interactions between intracellular signaling effectors and the TME are critical for sustained tumor growth, immune evasion, energy balance, and the survival of breast tumors. These interactions influence cellular signaling pathways and are pivotal in the progression, metastatic behavior, and variability of therapeutic responses in breast cancer (Table 2). This section reviews the signaling pathways and their cross-talk within breast tumors. While many of these pathways are not unique to breast cancer, they share oncogenic hubs across solid tumors. Here, we emphasize features and mechanisms specific to breast cancer.

**Table 2:** Key signaling pathways in breast cancer: Functions, TME interactions, and therapeutic approaches

**Pathway**	**Principal biological roles**	**TME-embedded interactions**	**Representative therapeutic strategies**	**References**
PI3K/AKT/mTOR	Cell survival; metabolism; growth; therapy resistance	CAF-derived IL-6/IGF-1; exosomal activation in immune and endothelial cells	PI3Kα inhibitors (alpelisib); AKT inhibitors; combinations with immune/TME modulators	Khorasani, 2024 [66]
TGF-β/SMAD	EMT; fibrosis; immune suppression; CAF differentiation	Drives ECM stiffening; recruits Tregs; influences CAF and macrophage phenotypes	TGF-β inhibitors and traps; dual blockade with PD-1/PD-L1 inhibitors	Luo, 2023 [69]
JAK/STAT (STAT3-centered)	Inflammation; stemness; immune evasion; cytokine dependence	IL-6/IL-10 from CAFs and TAMs reinforce tumor and stromal STAT3 activity	JAK1/2 inhibitors; direct STAT3 inhibitors; combined checkpoint blockade	Shao, 2021 [72]
MAPK/ERK	Proliferation; differentiation; stress adaptation	Activated by CAF/TAM growth factors (EGF, HGF); supports EMT with PI3K	MEK inhibitors; rational combinations (with PI3K or ER therapies)	Luo, 2023 [75]
Wnt/β-Catenin	Stemness; EMT; niche maintenance	CAF- or macrophage-derived WNT ligands; hypoxia enhances signaling	Porcupine inhibitors; targeted pathway modulators (limited by toxicity)	Nasser, 2021 [76]
Notch	Angiogenesis; cell fate; CSC maintenance	Tumor–endothelial contact; supports perivascular CSC niches	γ-secretase inhibitors; context-dependent combination therapy	Nasser, 2021 [76]
YAP/TAZ (Hippo pathway)	Mechanotransduction; ECM stiffness response; EMT and stemness	Activated by stiff ECM, integrin/FAK signaling, and CAF-driven rigidity	FAK inhibitors; YAP–TEAD interaction disruptors; stroma-softening strategies	Ortega, 2022, Mokhtari, 2023 [80, 81]
NF-κB	Inflammation; survival; therapy resistance	Drives cytokine-rich tumor–stromal loops; maintains chronic inflammatory TME	IKKβ/NF-κB modulators; inflammation-directed therapy; combinations to reduce resistance	Cao, 2024 [83]

Abbreviations: PI3K/AKT/mTOR: Phosphoinositide 3-kinase/protein kinase B/mechanistic target of rapamycin; CAF: Cancer-associated fibroblast; CSC: Cancer stem cell; ECM: Extracellular matrix; EMT: Epithelial–mesenchymal transition; ER: Estrogen receptor; FAK: Focal adhesion kinase; IGF-1: Insulin-like growth factor 1; IKKβ: I kappa B kinase beta; JAK/STAT: Janus kinase/signal transducer and activator of transcription; MEK: Mitogen-activated protein kinase kinase; NF-κB: Nuclear factor kappa B; PD-1/PD-L1: Programmed cell death protein 1/programmed death-ligand 1; PI3Kα: Phosphoinositide 3-kinase alpha; TAMs: Tumor-associated macrophages; TGF-β/SMAD: Transforming growth factor beta/SMAD; Wnt/β-catenin: Wingless-related integration site/beta-catenin; YAP/TAZ: Yes-associated protein/transcriptional co-activator with PDZ-binding motif.

### PI3K/Akt/mTOR pathway

**Figure 2.**
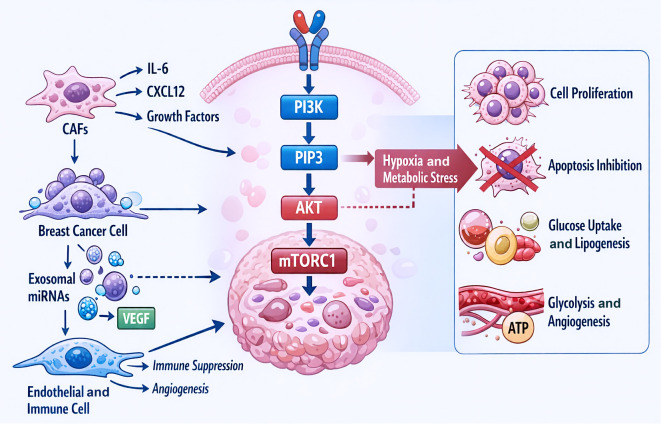
**PI3K/AKT/mTOR signaling pathway in breast cancer.** This diagram illustrates the activation of PI3K signaling by cancer-associated fibroblasts and other tumor cells in breast cancer. PI3K phosphorylates and converts PIP2 into PIP3, leading to AKT activation and subsequent mTORC1 signaling. Metabolic alterations and hypoxia enhance cell proliferation and survival by upregulating this pathway, which promotes glucose uptake, glycolysis, lipogenesis, and angiogenesis. Tumor-derived miRNAs and VEGF influence endothelial and immune cells within the breast tumor microenvironment, contributing to immune suppression and neovascularization. Abbreviations: AKT: Protein kinase B; ATP: Adenosine triphosphate; CAFs: Cancer-associated fibroblast(s); CXCL12: C-X-C motif chemokine ligand 12; IL-6: Interleukin-6; mTORC1: Mechanistic target of rapamycin complex 1; PI3K: Phosphoinositide 3-kinase; PIP3: Phosphatidylinositol (3,4,5)-trisphosphate; TME: Tumor microenvironment; VEGF: Vascular endothelial growth factor.

The activation of the altered phosphoinositide-3-kinase (PI3K)/AKT/mammalian target of rapamycin (mTOR) signaling axis enhances cellular proliferation, inhibits apoptosis, and promotes tumor progression, particularly in luminal and HER2-positive breast cancer subtypes (Figure 2). This pathway is initiated by PI3K activation. Sustained activation of PI3K/AKT/mTOR leads to metabolic alterations, including increased glucose uptake, lipogenesis, and enhanced cell survival. CAFs within breast tumors promote PI3K/AKT activation by releasing growth factors, such as IL-6 and CXCL12. Additionally, tumor-derived exosomal miRNAs and VEGF can activate PI3K in cancerous endothelial and immune cells, indirectly influencing immune suppression and angiogenesis. Furthermore, mTOR complex-1 (mTORC-1) serves as a critical integrator of nutrient and oxygen availability; hypoxia and metabolic stress induce HIF-1α activation, which modulates mTORC-1 activity and subsequently facilitates glycolysis and angiogenesis in the breast TME [66]. While PI3K/AKT/mTOR activation is frequently reported in solid tumors, breast cancer is characterized by a high prevalence of point mutations in the *PIK3CA*gene, particularly in estrogen receptor-positive subtypes [67, 68].

### TGF-β/SMAD pathway: Dual role in tumor suppression and promotion

Cancer-associated fibroblasts can release TGF-β within the TME, contributing to the TGF-β/SMAD signaling pathway, which regulates cell development and tissue homeostasis. This pathway is initiated when TGF-β binds to its serine/threonine kinase receptor, activating SMAD2/3 transcriptional complexes that translocate to the nucleus and regulate gene expression associated with cell-cycle arrest and EMT transcription factors (e.g., ZEB1, SNAIL). In the TME, TGF-β regulates fibroblast activation, ECM deposition, and immune system suppression. This pathway exhibits a bifunctional role in tumor biology; while TGF-β inhibits epithelial cell proliferation in early breast cancer stages, it promotes EMT, invasion, and metastasis in advanced stages. Continuous TGF-β elevation in the TME drives oncogenic signaling through two mechanisms: first, TGF-β induces CAF differentiation, contributing to ECM stiffness and activating the YAP/TAZ mechanotransduction pathway; second, TGF-β recruits Tregs and promotes M2 macrophage polarization, thereby inhibiting anti-tumor immunity within the TME [69]. Similar TGF-β-induced immune suppression has been reported in lung, pancreatic, and colorectal cancers, yet its intersection with mammary-specific CAFs and adipocytes contributes to ECM stiffness and high mammographic density in breast cancer [70, 71].

**Figure 3.**
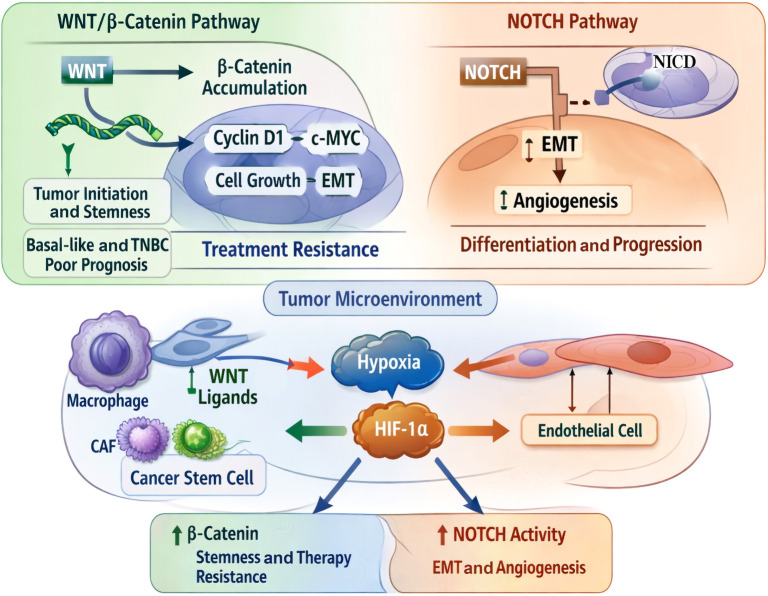
**Wnt/β-catenin and Notch signaling pathways in the breast tumor microenvironment**. This figure depicts the roles of Wnt/β-Catenin and Notch signaling in tumor initiation, stemness, EMT, angiogenesis, and therapy resistance within the breast tumor microenvironment. Wnt ligand secretion from stromal cells, such as macrophages and CAFs, facilitates β-catenin accumulation and the transcription of oncogenic targets, including *c-MYC* and Cyclin D1, thereby promoting tumor growth and stem-like characteristics, particularly in basal-like and triple-negative breast cancers. Simultaneously, Notch activation and the release of the Notch intracellular domain stimulate EMT, endothelial activation, and angiogenesis. Additionally, hypoxia-induced HIF-1α further enhances both pathways, thereby exacerbating cancer progression and treatment resistance. Abbreviations: *c-MYC*: Cellular MYC proto-oncogene; CAF: Cancer-associated fibroblast; Cyclin D1: G1/S-phase cell-cycle regulatory protein; EMT: Epithelial–mesenchymal transition; HIF-1α: Hypoxia-inducible factor 1 alpha; NICD: Notch intracellular domain; TNBC: Triple-negative breast cancer; WNT: Wingless-related integration site signaling ligands.

### JAK/STAT pathway: Inflammation and evasion

The Janus kinase/signaling transducer and activator of transcription (JAK/STAT) pathway is essential for mediating cytokine signaling in the breast TME. This pathway is initiated by the binding of cytokines (e.g., IL-6) or growth factors to their cell-surface receptors, activating JAKs and launching a phosphorylation cascade of STAT proteins. Phosphorylated STAT3 translocates to the nucleus, modulating transcriptional changes associated with cellular proliferation, invasion, and immune regulation. Within breast tumors, CAFs and macrophages can produce IL-6, intensifying STAT3 signaling in tumor cells. Continuous STAT3 activation induces both tumor and stromal cells to produce VEGF, matrix metalloproteinases, and PD-L1, exerting immunosuppressive and pro-inflammatory effects while promoting cancer stemness and chemoresistance. Furthermore, activated JAK/STAT signaling impedes dendritic cell maturation and T cytotoxic cell activation, contributing to immune evasion [72]. While this signaling pathway is activated by inflammatory cytokines in various tumors, in breast cancer, IL-6/STAT3 activation is particularly linked to obesity, chronic inflammation, and resistance to immune checkpoint inhibitors, especially in estrogen receptor-positive and TNBC patients [67, 73, 74].

### MAPK/ERK pathway

The mitogen-activated protein kinase (MAPK)/extracellular-signal-regulated kinase (ERK) pathway comprises a series of signaling cascades that connect extracellular activators (e.g., growth factors, mitogens, and hormones stimulating receptor tyrosine kinases) to transcriptional reprogramming, playing a pivotal role in breast tumor cell proliferation, survival, invasion, and metastasis. Within the breast TME, CAFs release growth factors, such as HGF and epidermal growth factor (EGF), sustaining MAPK activation in tumor cells. Conversely, cancer-associated immune cells produce tumorigenic cytokines, such as IL-1β and TNF-α, which promote ERK activation in cancer endothelial and immune cells. Continuous ERK activation induces PI3K/AKT, facilitating metabolic reprogramming, EMT, and cellular survival under stress conditions [75].

### Wnt/β-catenin and Notch pathway

The Wnt/β-Catenin and Notch signaling pathways are involved in tumor initiation, stemness, differentiation, progression, and treatment resistance (Figure 3). The Wnt/β-Catenin pathway regulates genes such as cyclin D1 and c-MYC, which are associated with cellular growth and EMT of tumor cells. An increase in cytoplasmic β-Catenin leads to its nuclear accumulation and transcriptional activation of oncogenic factors, such as c-MYC, observed in basal-like and TNBC patients, indicating poor prognosis and treatment resistance. Within the breast TME, macrophages and CAFs produce and release Wnt ligands, promoting tumor stem-like properties and increasing resistance to treatment. In contrast, tumor cells directly interact with stromal and endothelial cells within the TME, triggering Notch signaling that regulates EMT and angiogenesis in breast tumors. Hypoxic conditions in the TME enhance both Notch and Wnt signaling pathways, as HIF-1α increases the stability of the Notch intracellular domain, contributing to sustained Notch activity and increased β-Catenin signaling, which integrates metabolic stress with preserved cancer stemness [76]. In basal-like and TNBC, Wnt/β-Catenin and Notch signaling cooperate with CAFs and TAM to promote cancer stemness and metastasis [73, 77–79].

### YAP/TAZ (Hippo pathway): Mechanotransduction and ECM stiffness

Yes-associated protein (YAP) and transcriptional co-activator with PDZ-binding motif (TAZ) are key effectors of the Hippo pathway and mechanotransduction. In breast cancer, ECM stiffness and cytoskeletal tension inhibit the Hippo kinase cascade (LATS1/2-MST1/2), leading to nuclear translocation of YAP/TAZ, transcriptional activation of proliferative genes, and interaction with transcription factors to promote EMT, stemness, and therapy resistance. CAF-derived stiff ECM and integrin-FAK signaling sustain YAP/TAZ activity in both tumor and stromal compartments. Therapeutic targeting of YAP/TAZ is challenging due to their role as transcriptional co-activators rather than kinases; however, inhibitors disrupting YAP-TEAD interactions or modulating actin cytoskeleton tension have shown preclinical efficacy [80, 81]. YAP/TAZ mechanotransduction is activated by matrix stiffness in many solid tumors, but in breast cancer, it promotes obesity-associated inflammation, ECM remodeling, and increased matrix stiffness [70, 82].

### NF-κB pathway: Inflammation and resistance

NF-κB is a central regulator of inflammatory and stress responses, linking oncogenic signaling to immune modulation. Constitutive NF-κB activation in breast cancer cells drives the transcription of pro-inflammatory cytokines (IL-6 and IL-8), anti-apoptotic genes (*BCL2* and *XIAP*), and EMT inducers. Within the TME, NF-κB activation in macrophages and fibroblasts sustains a pro-inflammatory environment that promotes tumor progression and therapeutic resistance. Cytokine-mediated feedback loops involving TNF-α, IL-1β, and TGF-β perpetuate NF-κB signaling across tumor and stromal compartments. Moreover, NF-κB contributes to resistance to endocrine therapy and chemotherapy by inhibiting apoptosis and enhancing DNA-damage repair mechanisms [83]. In breast tumors, NF-κB signaling is closely associated with estrogen signaling, adipokines, and TAM-rich microenvironment, contributing to endocrine resistance, obesity, and poor clinical outcomes [67, 73].

### Interplay between TME and cancer stem cells

The self-renewal capacity of cancer stem cells (CSCs), along with their potential for tumor initiation and treatment resistance, significantly contributes to tumor heterogeneity, metastasis, and recurrence in breast cancer [84]. TME produces biomolecules that support cancer stemness, CSC maintenance, and plasticity through reciprocal interactions, establishing a dynamic feedback loop. CAFs activate Wnt-β-Catenin, Notch, and YAP/TAZ signaling by releasing TGF-β, IL-6, and HGF, thereby expanding the CSC pool and promoting EMT. TAMs enhance CSC survival under hypoxia or nutrient deprivation by releasing cytokines (e.g., IL-6 and TNF-α) and tumor-derived exosomes that reinforce STAT3 and NF-κB signaling. Endothelial cells within perivascular niches provide Notch ligands, such as Jagged canonical Notch ligand 1 (JAG1), and soluble factors that sustain CSC quiescence and metastatic potential. Hypoxic gradients, mediated by HIF-1α, further upregulate CSC markers such as *CD44*, Aldehyde Dehydrogenase 1 Family Member A1 (*ALDH1*), and SRY-Box Transcription Factor 2 (*SOX2*), thereby linking metabolic stress to stemness. Conversely, CSCs actively reprogram the TME to their advantage. CSC-derived exosomes deliver miRNAs (e.g., *miR-21*, *miR-181*) that polarize TAMs toward an M2-like immunosuppressive phenotype and activate CAFs, thereby amplifying ECM stiffness via Lysyl oxidases (LOX/LOXL2) [85, 86]. CSCs also secrete Wnt ligands and Osteopontin to recruit immunosuppressive MDSCs and Tregs, fostering an immune-evasive niche [85, 87, 88]. Recent evidence has highlighted how these interactions confer therapeutic resistance. For instance, Li et al. demonstrated that breast CSCs exploit CAF-derived CXCL12/CXCR4 signaling to evade chemotherapy, while TAM-secreted Periostin stabilizes YAP/TAZ in CSCs, promoting dormancy and recurrence [84]. This bidirectional CSC-TME crosstalk underscores the need for combined therapies targeting both compartments, such as Notch inhibitors with CAF-modulating agents or exosome blockers alongside checkpoint inhibitors, to eradicate the CSC reservoir and overcome resistance [89, 90].

## Therapeutic Targeting of TME–Signaling Interactions

The recognition that breast cancer progression is governed not only by tumor-intrinsic genetic alterations but also by the surrounding microenvironment has prompted the development of therapeutic strategies that target the TME through the interconnection of signaling networks [91]. Combination approaches that concurrently modulate stromal, immune, and molecular pathways are emerging as promising paradigms in precision oncology.

### TME-targeted strategies

Both CAFs and the ECM are central regulators of tumor stiffness, invasion, and immune exclusion. Pharmacologic strategies aimed at inhibiting CAF activation or ECM cross-linking have been proposed to normalize the stroma and enhance therapeutic delivery. Galunisertib and vactosertib target the TGF-β pathway, suppressing myofibroblast differentiation and collagen deposition, thereby improving immune cell infiltration [92]. Inhibitors of Lysyl oxidases (LOX/LOXL2), such as simtuzumab and PXS-5153A, reduce matrix stiffness and metastatic potential [93]. Additionally, blockade of fibroblast activation protein (FAP) expressed on CAFs has demonstrated preclinical efficacy, particularly when combined with immune checkpoint inhibitors [94]. Anti-angiogenic agents are well-established medications targeting endothelial cell-driven hypoxia and perivascular CSC niches, where Notch and Wnt signaling are involved in breast cancer management [42, 76, 77, 95]. Bevacizumab, an anti-VEGF-A monoclonal antibody, was the first approved anti-angiogenic agent, demonstrating improvements in progression-free survival, although without a corresponding benefit in overall survival [96].

Emerging evidence indicates that partial vessel normalization, rather than complete inhibition, optimizes immune cell trafficking, reprograms the hypoxic TME, improves CSC sensitivity to medications, and promotes drug delivery [97]. Combining angiogenesis inhibitors with immune checkpoint blockade enhances therapeutic efficacy by alleviating hypoxia and reprogramming the immune microenvironment. Lenvatinib, an oral tyrosine kinase inhibitor, in combination with pembrolizumab, an immune checkpoint inhibitor, has produced durable responses in subsets of metastatic TNBC [97]. Additionally, novel multi-target tyrosine kinase inhibitors (TKIs) are under clinical investigation for their combined anti-angiogenic and immunomodulatory effects [98–100]. For instance, Peng et al. introduced tinengotinib, a multi-TKI agent effective in treating patients with TNBC by targeting Aurora A/B, VEGFRs, FGFR 1/2/3, and JAK 1/2, ultimately inducing upregulation of CXCL10/11 or reducing tumor-associated macrophage infiltration [101].

The advent of immunotherapy has transformed cancer treatment; however, only a subset of breast cancer patients, predominantly those with TNBC, achieve durable clinical benefits. The immunosuppressive architecture of the TME is a key contributor to therapeutic resistance [102]. Immune checkpoint inhibitors targeting PD-1/PD-L1 (e.g., pembrolizumab and atezolizumab) and CTLA-4 (e.g., ipilimumab) restore cytotoxic T-cell activity, particularly when administered in combination with chemotherapy or radiotherapy [103, 104].

### Targeting CSC-TME Interaction

CSCs and TME crosstalk, particularly in hypoxic perivascular niches, plays a pivotal role in cancer progression, metastasis, and resistance by interfering with signaling pathways such as Notch, Wnt/β-Catenin, and YAP/TAZ. Endothelial-derived JAG1 activates Notch signaling in CSCs, promoting chemoresistance [105]. Gamma-secretase inhibitors, such as AL101, disrupt Notch signaling in TNBC patients [106]. Wnt ligands from CAFs and macrophages sustain CSC self-renewal; porcupine inhibitors, such as RXC004, can effectively reduce tumor initiation, suppress tumor growth, and reverse immune evasion in cancer models [95]. YAP/TAZ-TEAD disruptors (e.g., VT3989) target mechanotransduction from stiff ECM to CSC stemness, showing promise in breast cancer treatment [82]. Exosomes mediate CSC-TME communication by transferring non-coding RNAs, biomolecules, or therapeutic agents [107–109]. Exosome inhibitors, such as GW4869, are effective in inhibiting angiogenesis and sensitizing tumor cells to chemotherapeutic agents [110].

### Signaling pathway targeted strategies

Given the extensive role of signaling cascades in the TME, pathway-targeted inhibitors constitute a major therapeutic strategy. Alpelisib, a PI3Kα-selective inhibitor, in combination with fulvestrant, an estrogen receptor downregulator, improves clinical outcomes in *PIK3CA*-mutant ER-positive breast cancer patients [111]. Capivasertib, an AKT inhibitor, has demonstrated progression-free survival benefits when administered alongside endocrine therapy [112].

Nevertheless, stromal and immune-derived feedback mediated by factors such as IL-6, insulin-like growth factor 1 (IGF-1), and CXCL12 can reactivate PI3K signaling. In TNBC, dual inhibition of PI3K and immune checkpoints enhances anti-tumor immunity by reducing PD-L1 expression and limiting MDSC recruitment [113].

The MAPK/ERK signaling cascade drives proliferation and invasion, particularly in HER2-positive and basal-like breast cancer subtypes. MEK inhibitors, including trametinib and selumetinib, demonstrate modest single-agent activity but exhibit synergistic effects when combined with PI3K or ER inhibitors [114]. The TGF-β/SMAD pathway represents a key target for stromal normalization and immunomodulation. Small-molecule inhibitors, such as galunisertib and vactosertib, as well as neutralizing antibodies, such as fresolimumab, reduce CAF activation and promote immune cell infiltration [115].

### Combination and nanotechnology-based therapies

Monotherapies frequently fail due to pathway redundancy and adaptive resistance. Rational combination strategies aim to simultaneously target tumor-cell signaling and microenvironmental components. The integration of multi-omics and spatial transcriptomics has enabled the precise mapping of pathway activation within the tumor microenvironment, thereby informing personalized combination therapies [116]. Exosome-based nanocarriers, engineered to deliver small interfering RNA (siRNAs) or inhibitors targeting STAT3, PI3K, or TGF-β, demonstrate enhanced bioavailability and reduced off-target toxicity [117]. In preclinical breast cancer models, exosome-mediated delivery of a pre-designed siRNA inhibited PI3K/AKT/mTOR signaling in the MDA-MB-231 breast cancer cell line by silencing the *PIK3CA* gene, significantly reducing cell viability and migration capacity [118]. Smart nanoparticles responsive to pH, redox potential, or enzymatic activity within the tumor microenvironment allow for spatiotemporal drug release, maximizing therapeutic efficacy while sparing normal tissue [119].

## Discussion and future directions

Despite significant advancements in understanding the dynamic interplay between the TME and molecular signaling in breast cancer, substantial challenges remain in translating these insights into durable clinical benefits. The TME is neither static nor homogeneous; it evolves spatiotemporally in response to therapeutic pressure, presenting a moving target for intervention. Future research should prioritize addressing this complexity through advanced preclinical modeling, precise biomarker identification, and the development of integrative, multimodal therapeutic strategies.

Breast tumors exhibit extensive intratumoral heterogeneity at genetic, epigenetic, and microenvironmental levels. Single-cell and spatial multi-omics analyses have identified diverse subpopulations of CAFs, immune cells, and endothelial cells, which dynamically remodel in response to therapy [116]. Temporal profiling indicates that interventions such as chemotherapy, endocrine therapy, or immunotherapy rapidly reconfigure cytokine networks and signaling hierarchies, generating drug-tolerant states. To capture this dynamic, longitudinal sampling approaches, including liquid biopsies and serial tissue biopsies in combination with spatial transcriptomics, can effectively monitor real-time TME evolution [120]. Computational modeling and AI-driven network inference are crucial for predicting adaptive resistance trajectories and optimizing combination therapy schedules [121]. Integrating these insights into *in vivo* lineage-tracing and organoid systems allows for mechanistic validation and time-resolved mapping of signaling feedback loops.

A major challenge in TME-directed therapy is the lack of predictive biomarkers to identify patient subsets most likely to respond. Current stratification strategies predominantly rely on genetic alterations (e.g., *PIK3CA* and *BRCA1/2*) rather than stromal or immune signatures. The integration of multi-omics and spatial data offers opportunities to develop composite biomarkers that encompass CAF activation states, immune infiltration patterns, ECM stiffness, and cytokine landscapes. For instance, circulating exosomes enriched with specific miRNAs or proteins, such as PD-L1 and lysyl oxidase-like 2 (LOXL2), represent promising minimally invasive biomarkers of TME remodeling and therapeutic responses [86, 122]. Dinca et al. investigated the synergistic effects of high co-expression of oncostatin M (OSM), a pro-inflammatory cytokine that induces EMT, and *LOXL-2* in patients with invasive ductal carcinoma of the breast, which is associated with a worse rate of metastasis and reduced survival [86]. In the study by Liu et al., the evaluation of tumor cell surface and exosomal PD-L1 expression levels was introduced as an important biomarker for targeted therapy in cancer management [122]. Implementing these multidimensional biomarkers in clinical trials will necessitate standardized protocols for sample collection, data normalization, and analytical workflows.

Breast cancer progression reflects the culmination of a complex and dynamic interplay between tumor-intrinsic molecular signaling and the surrounding microenvironment. The TME, comprising fibroblasts, immune and endothelial cells, adipocytes, ECM, and soluble mediators, acts both as an accomplice and adversary, shaping tumor evolution, therapeutic response, and metastatic potential. Over the past decade, integrative research has revealed how signaling pathways, including PI3K/AKT/mTOR, TGF-β/SMAD, JAK/STAT, MAPK/ERK, Wnt/β-Catenin, Notch, YAP/TAZ, and NF-κB, interface with TME-derived cues to drive hallmark processes such as EMT, angiogenesis, immune evasion, and therapy resistance. These interactions are highly interconnected rather than linear, forming a network of feedback and redundancy that confers remarkable plasticity and resilience to tumor ecosystems. Translational advances, including TME-targeted therapies, immune checkpoint blockade, pathway-specific inhibitors, and nanotechnology-based co-delivery systems, underscore the therapeutic potential of disrupting this crosstalk. Nevertheless, the intrinsic heterogeneity and temporal evolution of the TME continue to limit durable responses. Emerging 3D organoid and microfluidic platforms, combined with single-cell and spatial multi-omics, now allow for unprecedented mapping of these interactions with spatial and temporal precision. The next frontier in breast oncology lies in ecosystem-level approaches: integrating molecular, spatial, and computational insights to design adaptive, personalized interventions. Artificial intelligence and predictive modeling further bridge experimental and clinical data, enabling real-time monitoring and optimization of therapeutic strategies.

## Conclusion

In conclusion, targeting the breast cancer microenvironment and its signaling circuitry has transitioned from a peripheral consideration to a central pillar of modern oncology. By conceptualizing the TME as an evolving ecosystem and leveraging next-generation analytical and therapeutic tools, future research promises to move beyond incremental improvements toward true disease reprogramming and sustained remission.

## Data Availability

This was a review article and no new data were generated.

## References

[1] Wilkinson L, Gathani T (2022). Understanding breast cancer as a global health concern. British Journal of Radiology.

[2] Szymiczek A, Lone A, Akbari MR (2021). Molecular intrinsic versus clinical subtyping in breast cancer: a comprehensive review. Clinical Genetics.

[3] Xiao Y, Yu T-J, Xu Y, Ding R, Wang Y-P, Jiang Y-Z (2023). Emerging therapies in cancer metabolism. Cell Metabolism.

[4] Liu B, Zhou H, Tan L, Siu KTH, Guan X-Y (2024). Exploring treatment options in cancer: tumor treatment strategies. Signal Transduction and Targeted Therapy.

[5] Farc O, Cristea V (2021). An overview of the tumor microenvironment, from cells to complex networks (Review). Experimental and Therapeutic Medicine.

[6] Li JJ, Tsang JY, Tse GM (2021). Tumor microenvironment in breast cancer-Updates on therapeutic implications and pathologic assessment. Cancers.

[7] Panda VK, Mishra B, Mahapatra S, Swain B, Malhotra D, Saha S (2025). Molecular insights on signaling cascades in breast cancer: a comprehensive review. Cancers.

[8] Neophytou CM, Panagi M, Stylianopoulos T, Papageorgis P (2021). The role of tumor microenvironment in cancer metastasis: Molecular mechanisms and therapeutic opportunities. Cancers.

[9] Tan K, Naylor MJ (2022). Tumour microenvironment-Immune cell interactions influencing breast cancer heterogeneity and disease progression. Frontiers in Oncology.

[10] Akinsipe T, Mohamedelhassan R, Akinpelu A, Pondugula SR, Mistriotis P, Avila LA (2024). Cellular interactions in tumor microenvironment during breast cancer progression: new frontiers and implications for novel therapeutics. Frontiers in Immunology.

[11] Zhang W, Wang J, Liu C, Li Y, Sun C, Wu J (2023). Crosstalk and plasticity driving between cancer-associated fibroblasts and tumor microenvironment: significance of breast cancer metastasis. Journal of Translational Medicine.

[12] Hu D, Li Z, Zheng B, Lin X, Pan Y, Gong P (2022). Cancer-associated fibroblasts in breast cancer: Challenges and opportunities. Cancer Communications.

[13] Chen P-Y, Wei W-F, Wu H-Z, Fan L-S, Wang W (2021). Cancer-associated fibroblast heterogeneity: a factor that cannot be ignored in immune microenvironment remodeling. Frontiers in Immunology.

[14] Guo T, Xu J (2024). Cancer-associated fibroblasts: a versatile mediator in tumor progression, metastasis, and targeted therapy. Cancer and Metastasis Reviews.

[15] Jeong W, Lee SJ (2025). Engineering complexity: Advances in 3D breast cancer models for precision oncology. Advanced Healthcare Materials.

[16] Wu X, Lu W, Zhang W, Zhang D, Mei H, Zhang M (2023). Integrated analysis of single-cell RNA-seq and bulk RNA-seq unravels the heterogeneity of cancer-associated fibroblasts in TNBC. Aging (Albany NY).

[17] Jia H, Chen X, Zhang L, Chen M (2025). Cancer associated fibroblasts in cancer development and therapy. Journal of Hematology & Oncology.

[18] Desbois M, Wang Y (2021). Cancer-associated fibroblasts: Key players in shaping the tumor immune microenvironment. Immunological Reviews.

[19] Xu Q, Chen S, Hu Y, Huang W (2021). Landscape of immune microenvironment under immune cell infiltration pattern in breast cancer. Frontiers in Immunology.

[20] Jin J, Li Y, Zhao Q, Chen Y, Fu S, Wu J (2021). Coordinated regulation of immune contexture: crosstalk between STAT3 and immune cells during breast cancer progression. Cell Communication and Signaling.

[21] Huang X, Cao J, Zu X (2022). Tumor-associated macrophages: an important player in breast cancer progression. Thoracic Cancer.

[22] Tharp KM, Kersten K, Maller O, Timblin GA, Stashko C, Canale FP (2024). Tumor-associated macrophages restrict CD8+ T cell function through collagen deposition and metabolic reprogramming of the breast cancer microenvironment. Nature Cancer.

[23] Allison E, Edirimanne S, Matthews J, Fuller SJ (2023). Breast cancer survival outcomes and tumor-associated macrophage markers: a systematic review and meta-analysis. Oncology and Therapy.

[24] Xiao M, He J, Yin L, Chen X, Zu X, Shen Y (2021). Tumor-associated macrophages: Critical players in drug resistance of breast cancer. Frontiers in Immunology.

[25] Seung E, Xing Z, Wu L, Rao E, Cortez-Retamozo V, Ospina B (2022). A trispecific antibody targeting HER2 and T cells inhibits breast cancer growth via CD4 cells. Nature.

[26] Seif F, Torki Z, Zalpoor H, Habibi M, Pornour M (2023). Breast cancer tumor microenvironment affects Treg/IL-17-producing Treg/Th17 cell axis: Molecular and therapeutic perspectives. Molecular Therapy Oncolytics.

[27] Li T, Wu B, Yang T, Zhang L, Jin K (2020). The outstanding antitumor capacity of CD4+ T helper lymphocytes. Biochimica et Biophysica Acta (BBA) - Reviews on Cancer.

[28] Zareinejad M, Mehdipour F, Roshan-Zamir M, Faghih Z, Ghaderi A (2023). Dual functions of T lymphocytes in breast carcinoma: From immune protection to orchestrating tumor progression and metastasis. Cancers.

[29] Andreu-Sanz D, Kobold S (2023). Role and potential of different T helper cell subsets in adoptive cell therapy. Cancers.

[30] Silva RCMC, Lopes MF, Travassos LH (2023). Distinct T helper cell-mediated antitumor immunity: T helper 2 cells in focus. Cancer Pathogenesis and Therapy.

[31] Schmidt M, Weyer-Elberich V, Hengstler JG, Heimes A-S, Almstedt K, Gerhold-Ay A (2018). Prognostic impact of CD4-positive T cell subsets in early breast cancer: a study based on the FinHer trial patient population. Breast Cancer Research.

[32] Liu H, Wang Z, Zhou Y, Yang Y (2023). MDSCs in breast cancer: an important enabler of tumor progression and an emerging therapeutic target. Frontiers in Immunology.

[33] Wang H, Zhou F, Qin W, Yang Y, Li X, Liu R (2025). Metabolic regulation of myeloid-derived suppressor cells in tumor immune microenvironment: targets and therapeutic strategies. Theranostics.

[34] Virassamy B, Caramia F, Savas P, Sant S, Wang J, Christo SN (2023). Intratumoral CD8+ T cells with a tissue-resident memory phenotype mediate local immunity and immune checkpoint responses in breast cancer. Cancer Cell.

[35] Hasheminia A, Mann S, Liblik K, El-Diasty M (2025). The CCL17/CCL22-CCR4 axis in pain pathogenesis: a comprehensive review of immune-mediated mechanisms and therapeutic opportunities. Molecular Neurobiology.

[36] Toney NJ, Opdenaker LM, Cicek K, Frerichs L, Kennington CR, Oberly S (2022). Tumor-B-cell interactions promote isotype switching to an immunosuppressive IgG4 antibody response through upregulation of IL-10 in triple negative breast cancers. Journal of Translational Medicine.

[37] Kim SS, Sumner WA, Miyauchi S, Cohen EEW, Califano JA, Sharabi AB (2021). Role of B cells in responses to checkpoint blockade immunotherapy and overall survival of cancer patients. Clinical Cancer Research.

[38] Hu Q, Hong Y, Qi P, Lu G, Mai X, Xu S (2021). Atlas of breast cancer infiltrated B-lymphocytes revealed by paired single-cell RNA-sequencing and antigen receptor profiling. Nature Communications.

[39] Lopes MEF (2024). Unravel the role of pericytes in endothelial cell dysfunction induced by brain metastatic breast cancer cells [dissertation]. Coimbra (Portugal): Universidade de Coimbra;.

[40] Baumann J, Tsao C-C, Patkar S, Huang S-F, Francia S, Magnussen SN (2022). Pericyte, but not astrocyte, hypoxia inducible factor-1 (HIF-1) drives hypoxia-induced vascular permeability in vivo. Fluids and Barriers of the CNS.

[41] Thomas JA, Gireesh Moly AG, Xavier H, Suboj P, Ladha A, Gupta G (2023). Enhancement of immune surveillance in breast cancer by targeting hypoxic tumor endothelium: Can it be an immunological switch point?. Frontiers in Oncology.

[42] Akil A, Gutiérrez-García AK, Guenter R, Rose JB, Beck AW, Chen H (2021). Notch signaling in vascular endothelial cells, angiogenesis, and tumor progression: an update and prospective. Frontiers in Cell and Developmental Biology.

[43] Liu J, Gao F, Wang D, Zhou R, Huang C (2025). Effects and mechanisms of exosomes in microenvironment angiogenesis in breast cancer: an updated review. Oncological Research.

[44] Ritter A, Kreis N-N, Hoock SC, Solbach C, Louwen F, Yuan J (2022). Adipose tissue-derived mesenchymal stromal/stem cells, obesity and the tumor microenvironment of breast cancer. Cancers.

[45] Ritter A, Kreis N-N, Roth S, Friemel A, Safdar BK, Hoock SC (2023). Cancer-educated mammary adipose tissue-derived stromal/stem cells in obesity and breast cancer: spatial regulation and function. Journal of Experimental & Clinical Cancer Research.

[46] Bunnell BA, Martin EC, Matossian MD, Brock CK, Nguyen K, Collins-Burow B (2022). The effect of obesity on adipose-derived stromal cells and adipose tissue and their impact on cancer. Cancer and Metastasis Reviews.

[47] Brown KA (2021). Metabolic pathways in obesity-related breast cancer. Nature Reviews Endocrinology.

[48] Lin Z, Hua G, Hu X (2024). Lipid metabolism associated crosstalk: the bidirectional interaction between cancer cells and immune/stromal cells within the tumor microenvironment for prognostic insight. Cancer Cell International.

[49] Lue JC, Radisky DC (2025). From embryogenesis to senescence: the role of mammary gland physiology in breast cancer risk. Cancers.

[50] Wu C, Dong S, Huang R, Chen X (2023). Cancer-associated adipocytes and breast cancer: intertwining in the tumor microenvironment and challenges for cancer therapy. Cancers.

[51] Kristensen TB, Knutsson MLT, Wehland M, Laursen BE, Grimm D, Warnke E (2014). Anti-vascular endothelial growth factor therapy in breast cancer. International Journal of Molecular Sciences.

[52] Zhang M, Liu J, Liu G, Xing Z, Jia Z, Li J (2021). Anti-vascular endothelial growth factor therapy in breast cancer: Molecular pathway, potential targets, and current treatment strategies. Cancer Letters.

[53] Martinez J, Smith PC (2021). The dynamic interaction between extracellular matrix remodeling and breast tumor progression. Cells.

[54] Jahin I, Phillips T, Marcotti S, Gorey M-A, Cox S, Parsons M (2023). Extracellular matrix stiffness activates mechanosensitive signals but limits breast cancer cell spheroid proliferation and invasion. Frontiers in Cell and Developmental Biology.

[55] Ishihara S, Haga H (2022). Matrix stiffness contributes to cancer progression by regulating transcription factors. Cancers.

[56] Li Y, Ganesan K, Chen J (2022). Role of biological mediators of tumor-associated macrophages in breast cancer progression. Current Medicinal Chemistry.

[57] Amer HT, Stein U, El Tayebi HM (2022). The monocyte, a maestro in the tumor microenvironment (TME) of breast cancer. Cancers.

[58] Huang D, Lin D, Liang S, Lin J (2025). Expression of CXCR4 in the primary lesion of recurrent metastatic breast cancer and its association with prognosis. International Journal of General Medicine.

[59] Habanjar O, Bingula R, Decombat C, Diab-Assaf M, Caldefie-Chezet F, Delort L (2023). Crosstalk of inflammatory cytokines within the breast tumor microenvironment. International Journal of Molecular Sciences.

[60] Zhi S, Chen C, Huang H, Zhang Z, Zeng F, Zhang S (2024). Hypoxia-inducible factor in breast cancer: role and target for breast cancer treatment. Frontiers in Immunology.

[61] Corchado-Cobos R, García-Sancha N, Mendiburu-Eliçabe M, Gómez-Vecino A, Jiménez-Navas A, Pérez-Baena MJ (2022). Pathophysiological integration of metabolic reprogramming in breast cancer. Cancers.

[62] Adhikari S, Guha D, Mohan C, Mukherjee S, Tyler JK, Das C Reprogramming carbohydrate metabolism in cancer and its role in regulating the tumor microenvironment.

[63] Brena D, Huang M-B, Bond V (2022). Extracellular vesicle-mediated transport: Reprogramming a tumor microenvironment conducive with breast cancer progression and metastasis. Translational Oncology.

[64] Bertolini I, Perego M, Ghosh JC, Kossenkov AV, Altieri DC (2022). NFκB activation by hypoxic small extracellular vesicles drives oncogenic reprogramming in a breast cancer microenvironment. Oncogene.

[65] Peng B, Bartkowiak K, Song F, Nissen P, Schlüter H, Siebels B (2024). Hypoxia-induced adaptations of N-glycomes and proteomes in breast cancer cells and their secreted extracellular vesicles. International Journal of Molecular Sciences.

[66] Khorasani ABS, Hafezi N, Sanaei M-J, Jafari-Raddani F, Pourbagheri-Sigaroodi A, Bashash D (2024). The PI3K/AKT/mTOR signaling pathway in breast cancer: Review of clinical trials and latest advances. Cell Biochemistry and Function.

[67] Laskar TT, Laskar HM, Mazumder JA, Bhattacharjee R, Husain MI, Das B (2025). Decoding breast cancer: insights into molecular pathways and therapeutic approaches. Discover Oncology.

[68] Hamadeh LN, Farhat L, Hilal L, Assi H, Nasr F, Chahine G (2023). Frequency and mutational spectrum of PIK3CA gene mutations in breast cancer patients: Largest and first report from Lebanon. Gene.

[69] Luo D, Zeng X, Zhang S, Li D, Cheng Z, Wang Y (2023). Pirfenidone suppressed triple-negative breast cancer metastasis by inhibiting the activity of the TGF-β/SMAD pathway. Journal of Cellular and Molecular Medicine.

[70] Hawes ML, Moody MA, McCauley CR, Huddleston AG, Solanky M, Khosravi DH (2025). Oncogenic effects of ECM remodeling in obesity and breast cancer. Oncogene.

[71] Wu F, Yang J, Liu J, Wang Y, Mu J, Zeng Q (2021). Signaling pathways in cancer-associated fibroblasts and targeted therapy for cancer. Signal Transduction and Targeted Therapy.

[72] Shao F, Pang X, Baeg GH (2021). Targeting the JAK/STAT signaling pathway for breast cancer. Current Medicinal Chemistry.

[73] Malla RR, Kiran P (2022). Tumor microenvironment pathways: Cross regulation in breast cancer metastasis. Genes & Diseases.

[74] Ruan G-T, Zhu L-C, Xie H-L, Zhang H-Y, Song M-M, Deng L (2025). Adipocyte-derived IL6 and triple-negative breast cancer cell-derived CXCL1 co-activate STAT3/NF-κB pathway to mediate the crosstalk between adipocytes and triple-negative breast cancer cells. Cell Death Discovery.

[75] Liu Q, Liu Y, Li X, Wang D, Zhang A, Pang J (2023). Perfluoroalkyl substances promote breast cancer progression via ERα and GPER mediated PI3K/Akt and MAPK/Erk signaling pathways. Ecotoxicology and Environmental Safety.

[76] Nasser F, Moussa N, Helmy MW, Haroun M (2021). Dual targeting of Notch and Wnt/β-catenin pathways: Potential approach in triple-negative breast cancer treatment. Naunyn-Schmiedeberg’s Archives of Pharmacology.

[77] Chimento A, D’Amico M, Pezzi V, De Amicis F (2022). Notch signaling in breast tumor microenvironment as mediator of drug resistance. International Journal of Molecular Sciences.

[78] Sun MX, Zhu HC, Yu Y, Yao Y, Li HY, Feng FB (2025). Role of the Wnt signaling pathway in the complex microenvironment of breast cancer and prospects for therapeutic potential (Review). International Journal of Oncology.

[79] Zhang X, Wang X, Shi S, Guo D (2025). Decoding the mechanisms underlying breast cancer brain metastasis: paving the way for precision therapeutics. Biomarker Research.

[80] Ortega Á, Vera I, Diaz MP, Navarro C, Rojas M, Torres W (2022). The YAP/TAZ signaling pathway in the tumor microenvironment and carcinogenesis: Current knowledge and therapeutic promises. International Journal of Molecular Sciences.

[81] Mokhtari RB, Ashayeri N, Baghaie L, Sambi M, Satari K, Baluch N (2023). The Hippo pathway effectors YAP/TAZ-TEAD oncoproteins as emerging therapeutic targets in the tumor microenvironment. Cancers.

[82] Luo J, Zou H, Guo Y, Tong T, Chen Y, Xiao Y (2023). The oncogenic roles and clinical implications of YAP/TAZ in breast cancer. British Journal of Cancer.

[83] Cao Y, Yi Y, Han C, Shi B (2024). NF-κB signaling pathway in tumor microenvironment. Frontiers in Immunology.

[84] Li YR, Fang Y, Lyu Z, Zhu Y, Yang L (2023). Exploring the dynamic interplay between cancer stem cells and the tumor microenvironment: implications for novel therapeutic strategies. Journal of Translational Medicine.

[85] Aghapour SA, Torabizadeh M, Bahreiny SS, Saki N, Jalali Far MA, Yousefi-Avarvand A (2024). Investigating the dynamic interplay between cellular immunity and tumor cells in the fight against cancer: an updated comprehensive review. IJBC.

[86] Dinca SC, Greiner D, Weidenfeld K, Bond L, Barkan D, Jorcyk CL (2021). Novel mechanism for OSM-promoted extracellular matrix remodeling in breast cancer: LOXL2 upregulation and subsequent ECM alignment. Breast Cancer Research.

[87] Yan Z, Hu X, Tang B, Deng F (2023). Role of osteopontin in cancer development and treatment. Heliyon.

[88] de Sousa EMF, Vermeulen L (2016). Wnt signaling in cancer stem cell biology. Cancers (Basel).

[89] Shen Y, Sun Y, Li X, Wang Y, Huang T, Li T (2026). Novel anti-tumor strategies: targeting the crosstalk between cancer stem cells and cancer-associated fibroblasts to resist drug resistance. Cancer Drug Resistance.

[90] Müller L, Tunger A, Plesca I, Wehner R, Temme A, Westphal D (2020). Bidirectional crosstalk between cancer stem cells and immune cell subsets. Frontiers in Immunology.

[91] Nwabo Kamdje AH, Seke Etet PF, Vecchio L, Muller JM, Krampera M, Lukong KE (2014). Signaling pathways in breast cancer: Therapeutic targeting of the microenvironment. Cellular Signalling.

[92] Kim B-G, Malek E, Choi SH, Ignatz-Hoover JJ, Driscoll JJ (2021). Novel therapies emerging in oncology to target the TGF-β pathway. Journal of Hematology & Oncology.

[93] Chopra V, Sangarappillai RM, Romero-Canelón I, Jones AM (2020). Lysyl oxidase like-2 (LOXL2): an emerging oncology target. Advanced Therapeutics.

[94] Fang J, Xiao L, Joo K-I, Liu Y, Zhang C, Liu S (2016). A potent immunotoxin targeting fibroblast activation protein for treatment of breast cancer in mice. International Journal of Cancer.

[95] Phillips C, Bhamra I, Eagle C, Flanagan E, Armer R, Jones CD (2022). The Wnt pathway inhibitor RXC004 blocks tumor growth and reverses immune evasion in Wnt ligand-dependent cancer models. Cancer Research Communications.

[96] Janes PW, Parslow AC, Cao D, Rigopoulos A, Lee F-T, Gong SJ (2024). An anti-VEGF-B antibody reduces abnormal tumor vasculature and enhances the effects of chemotherapy. Cancers.

[97] Chung HC, Saada-Bouzid E, Longo F, Yanez E, Im S-A, Castanon E (2024). Lenvatinib plus pembrolizumab for patients with previously treated, advanced, triple-negative breast cancer: Results from the triple-negative breast cancer cohort of the phase 2 LEAP-005 Study. Cancer.

[98] Santoni M, Iacovelli R, Colonna V, Klinz S, Mauri G, Nuti M (2021). Antitumor effects of the multi-target tyrosine kinase inhibitor cabozantinib: a comprehensive review of the preclinical evidence. Expert Review of Anticancer Therapy.

[99] Le Du F, Diéras V, Curigliano G (2021). The role of tyrosine kinase inhibitors in the treatment of HER2+ metastatic breast cancer. European Journal of Cancer.

[100] Mehta K, Hegde M, Girisa S, Vishwa R, Alqahtani MS, Abbas M (2024). Targeting RTKs/nRTKs as promising therapeutic strategies for the treatment of triple-negative breast cancer: evidence from clinical trials. Military Medical Research.

[101] Peng P, Qiang X, Li G, Li L, Ni S, Yu Q (2023). Tinengotinib (TT-00420), a novel spectrum-selective small-molecule kinase inhibitor, is highly active against triple-negative breast cancer. Molecular Cancer Therapeutics.

[102] Vuletic A, Mirjacic Martinovic K, Jurisic V (2025). The role of tumor microenvironment in triple-negative breast cancer and its therapeutic targeting. Cells.

[103] Singh S, Numan A, Maddiboyina B, Arora S, Riadi Y, Md S (2021). The emerging role of immune checkpoint inhibitors in the treatment of triple-negative breast cancer. Drug Discovery Today.

[104] Villacampa G, Navarro V, Matikas A, Ribeiro JM, Schettini F, Tolosa P (2024). Neoadjuvant immune checkpoint inhibitors plus chemotherapy in early breast cancer: a systematic review and meta-analysis. JAMA Oncology.

[105] Qiao X, Ma B, Sun W, Zhang N, Liu Y, Jia L (2022). JAG1 is associated with the prognosis and metastasis in breast cancer. Scientific Reports.

[106] Broner EC, Alpert G, Gluschnaider U, Mondshine A, Solomon O, Sloma I (2019). AL101 mediated tumor inhibition in notch-altered TNBC PDX models. Journal of Clinical Oncology.

[107] Xie F, Zhou X, Su P, Li H, Tu Y, Du J (2022). Breast cancer cell-derived extracellular vesicles promote CD8+ T cell exhaustion via TGF-β type II receptor signaling. Nature Communications.

[108] Li Q (2022). Role of exosomes in cellular communication between tumor cells and the tumor microenvironment. Oncology Letters.

[109] Wang S, Shu J, Wang N, He Z (2025). Exosomal non-coding RNAs: mediators of crosstalk between cancer and cancer stem cells. Cell Death Discovery.

[110] He J, He F, Yang Q, Li Q (2025). Blockade of exosome release sensitizes breast cancer to doxorubicin via inhibiting angiogenesis. Cancer Medicine.

[111] André F, Ciruelos EM, Juric D, Loibl S, Campone M, Mayer IA (2021). Alpelisib plus fulvestrant for PIK3CA-mutated, hormone receptor-positive, human epidermal growth factor receptor-2-negative advanced breast cancer: final overall survival results from SOLAR-1. Annals of Oncology.

[112] Luboff AJ, DeRemer DL (2024). Capivasertib: a novel AKT inhibitor approved for hormone-receptor-positive, HER-2-negative metastatic breast cancer. Annals of Pharmacotherapy.

[113] Chen X, Feng L, Huang Y, Wu Y, Xie N (2023). Mechanisms and strategies to overcome PD-1/PD-L1 blockade resistance in triple-negative breast cancer. Cancers.

[114] Britten CD (2013). PI3K and MEK inhibitor combinations: examining the evidence in selected tumor types. Cancer Chemotherapy and Pharmacology.

[115] Jing H, Gao Y, Jing L, Yang H, Liu S (2025). Recent advances in therapeutic use of transforming growth factor-beta inhibitors in cancer and fibrosis. Frontiers in Oncology.

[116] Ma A, Xiang L, Yuan J, Wang Q, Zhao L, Yan H (2025). Spatial transcriptomics decodes breast cancer microenvironment heterogeneity: From multidimensional dynamic profiling to precision therapy blueprint construction. Biomolecules.

[117] Zhu L, Qu J, Tian Q, Qin S, Xu Z, Zhang J (2026). Current research of novel nano-delivery carriers based on exosomes: preparation, targeted enhancement, delivery mechanism and clinical application. Journal of Materials Chemistry B..

[118] Silva R, Ferreira D, Rodrigues LR (2022). Exosome-based delivery of RNAi leads to breast cancer inhibition. Journal of Drug Delivery Science and Technology.

[119] Luo X, Zhang Q, Chen H, Hou K, Zeng N, Wu Y (2022). Smart nanoparticles for breast cancer treatment based on the tumor microenvironment. Frontiers in Oncology.

[120] Rossi M, Radisky DC (2024). Multiplex digital spatial profiling in breast cancer research: State-of-the-art technologies and applications across the translational science spectrum. Cancers.

[121] Mao Y, Shangguan D, Huang Q, Xiao L, Cao D, Zhou H (2025). Emerging artificial intelligence-driven precision therapies in tumor drug resistance: recent advances, opportunities, and challenges. Molecular Cancer.

[122] Liu J, Peng X, Yang S, Li X, Huang M, Wei S (2022). Extracellular vesicle PD-L1 in reshaping tumor immune microenvironment: biological function and potential therapy strategies. Cell Communication and Signaling.

[123] Markowitz J, Wesolowski R, Papenfuss T, Brooks TR, Carson WE 3rd (2013). Myeloid-derived suppressor cells in breast cancer. Breast Cancer Research and Treatment.

[124] Olkhanud PB, Damdinsuren B, Bodogai M, Gress RE, Sen R, Wejksza K (2011). Tumor-evoked regulatory B cells promote breast cancer metastasis by converting resting CD4+ T cells to T-regulatory cells. Cancer Research.

[125] Ehlers FAI, Blise KE, Betts CB, Sivagnanam S, Kooreman LFS, Hwang ES (2025). Natural killer cells occupy unique spatial neighborhoods in HER2-- and HER2+ human breast cancers. Breast Cancer Research.

[126] Chen S, Zhu H, Jounaidi Y (2024). Comprehensive snapshots of natural killer cells functions, signaling, molecular mechanisms and clinical utilization. Signal Transduction and Targeted Therapy.

[127] Wang Y, Li C, Li Y, Zhu Z (2017). Involvement of breast cancer stem cells in tumor angiogenesis. Oncology Letters.

[128] Kim J.

[129] El Alaa RSA, Al-Mannai W, Darwish N, Al-Mansoori L (2024). Adipose-derived stromal cells and cancer-associated fibroblasts: interactions and implications in tumor progression. International Journal of Molecular Sciences.

